# Using Low-Intensity Focused Ultrasound to Treat Depression and Anxiety Disorders: A Review of Current Evidence

**DOI:** 10.3390/brainsci15101129

**Published:** 2025-10-21

**Authors:** Ao Du, Manli Huang, Zheng Wang, Hetong Zhou, Huilong Duan, Shaohua Hu, Yinfei Zheng

**Affiliations:** 1 College of Biomedical Engineering and Instrument Science, Zhejiang University, Hangzhou 310007, China; duao2021phd@zju.edu.cn (A.D.); duanhl@zju.edu.cn (H.D.); 2 Department of Psychiatry, The First Affiliated Hospital, Zhejiang University School of Medicine, Hangzhou 310003, China; huangmanli@zju.edu.cn (M.H.); wangzheng1109@zju.edu.cn (Z.W.); 1168600035@zju.edu.cn (H.Z.); 3 Zhejiang Key Laboratory of Precision Psychiatry, Hangzhou 310003, China; 4 Nanhu Brain-Computer Interface Institute, Hangzhou 311100, China

**Keywords:** transcranial focused ultrasound stimulation, low intensity focused ultrasound, affective disorders, mood effect, depression, anxiety

## Abstract

**Background**: Depression and anxiety disorders impact millions globally. In recent years, low-intensity focused ultrasound (LIFU), characterized by its high precision, deep penetration, and non-invasive nature, has garnered significant interest in neuroscience and clinical practice. To enhance understanding of its effects on mood, therapeutic availability in treatment of depression/anxiety disorders, and potential mechanisms, a systematic review of studies investigating the emotional impact of LIFU on depressive/anxious-like animal models, healthy volunteers, and patients with depression or anxiety disorders has been undertaken. **Methods**: Relevant papers published before 15 July 2025 were searched across four databases: Web of Science, PubMed, Science Direct, and Embase. A total of 28 papers which met the inclusion and exclusion criteria are included in this review. **Results**: Our findings indicate that LIFU reversed the depressive/anxious-like behaviors in the animal models and showed antidepressant/anti-anxiety effects among the state-of-art clinical studies. For example, immobility time in FST or TST is reduced in depressive animal models, and HRSD/BAI scales are improved in human studies. Key molecules such as BDNF/5-HT are found restored in animal models, and FC between key brain areas related to depression/anxiety is modulated after LIFU treatment. Notably, no brain tissue damage was observed in animal studies, and only mild adverse effects (such as dizziness and vomiting) were noted in a few human studies. **Conclusions**: The studies using LIFU to treat depression and anxiety remain in the preliminary stage. The mechanisms underlying LIFU’s mood effects—such as activation or inhibition of specific brain regions or neural circuits, anti-inflammatory effects, alterations in functional connectivity, synaptic plasticity, neurotransmitter levels, and BDNF—remain incompletely understood and warrant further investigation. Nevertheless, the LIFU technique holds promise for regulating both cortical and subcortical brain areas implicated in depression/anxiety disorders as a precise neuromodulation tool.

## 1. Introduction

Mood disorders, primarily depression and bipolar disorders as classified by the DSM-5, affect more than 360 million people globally and are identified by the World Health Organization (WHO) as a significant inducement to morbidity and mortality [1,2]. Depression and anxiety disorders remained the leading causes of global burden diseases [3]. Traditional treatments for depression and anxiety disorders, including pharmacotherapy, psychotherapy, and physiotherapy, often yield suboptimal results or adverse effects, prompting treatment discontinuation [4,5,6,7]. Neuromodulation techniques have emerged as promising alternatives for such patients. Deep brain stimulation (DBS), as an invasive method, entails implanting electrodes within limbic system structures, such as the thalamus, hippocampus, and amygdala, to directly modulate brain activity [8,9]. However, challenges, such as invasiveness, long-term power requirements, and biocompatibility issues, limit its clinical acceptance [10,11]. In contrast, traditional non-invasive neuromodulation therapies, like transcranial direct current stimulation (tDCS) and transcranial magnetic stimulation (TMS), generally target the cerebral cortex to indirectly influence subcortical areas and modulate brain functions [12,13]. The side effects of these methods remain low but may be limited in their use due to their low spatial resolution and permeability depth in the brain [14]. In 2017, an innovative millimeter-scale, non-invasive electric deep brain temporally interfering stimulation method was proposed [15]. However, its application remains limited and in the early stages of development.

In recent years, low-intensity focused ultrasound (LIFU) has garnered attention as a promising non-invasive neuromodulation therapy [16,17,18,19,20]. The energy of focused ultrasound can reach the deep brain area beneath the skull with a high spatial resolution [21,22,23,24,25,26,27,28,29]. While ultrasound’s neuromodulatory effects have been recognized since the 1930s [30], its potential to impact mood function started to appear when Hameroff utilized transcranial ultrasound stimulation (TUS) on patients with chronic pain in 2013 [31]. Notably, mood improvements were observed following TUS compared to the placebo group. Despite the use of a diagnostic ultrasound system rather than a focused ultrasound device, this pilot study provided initial evidence of low-intensity ultrasound’s mood-altering capabilities. Building upon evidence suggesting LIFU’s capacity to increase brain-derived neurotrophic factor (BDNF) levels [32] and promote neurogenesis [33], its potential application in alleviating depressive symptoms was postulated [34]. While previous studies have touched upon LIFU’s emotional effects and preclinical/clinical research on depression and anxiety disorders [35,36,37,38,39,40,41,42,43], systematic reviews incorporating the latest discoveries are lacking.

**Figure 1:**
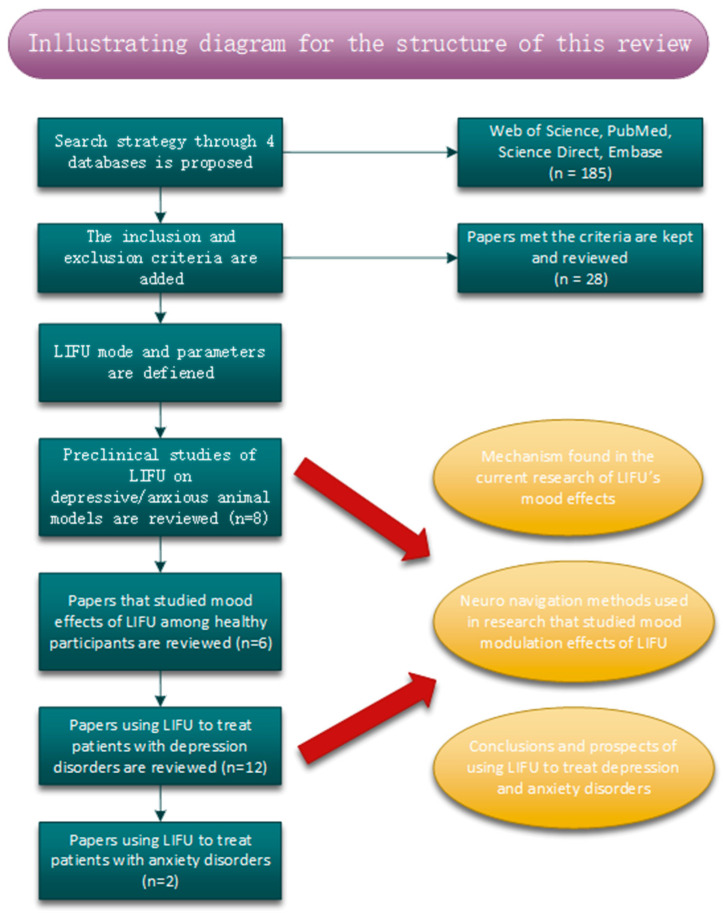
Illustrating diagram for the structure of this review.

This review aims to comprehensively summarize the current research on LIFU’s mood effects and antidepression/anxiety research across animal models and preclinical and clinical studies, including technique advancements, parameters, stimulation targets, mechanisms, treatment protocols, safety considerations, and potential limitations. By doing so, this review seeks to illuminate future research directions, providing deeper insights into the antidepression/anxiety mechanisms of LIFU and inspiring the development of non-invasive therapeutics for affective disorders. The illustrating diagram for the structure of this review is demonstrated in Figure 1.

## 2. Materials and Methods

### 2.1. Search Strategy

Papers published before 15 July 2025 from the following databases were searched: Web of Science, PubMed, Science Direct, and Embase. The search strategy incorporated a combination of the following keywords: “ultrasound stimulation”, “ultrasound neuromodulation”, “ transcranial pulse stimulation”, “theta burst transcranial ultrasound”, “low intensity focused ultrasound”, “low intensity pulsed ultrasound”, “transcranial ultrasound”, “transcranial focused ultrasound”, “low intensity ultrasound”, “low intensity pulsed ultrasound”, “LIFU”, “tFUS”, “TUS”, “TPS”, “LIPUS”, “affective disorder$”, “mood disorder$”, “emotion*”, “affective”, “depress*”, “dysthymic disorder”, “dysthymia”, “bipolar disorder”, “cyclothymic disorder”, “mania”, “manic”, “anxiety”, “antianxiety”, and ”antidepress*”.

Additionally, reference lists of identified publications were also checked to include potentially relevant studies.

### 2.2. Inclusion and Exclusion Criteria

The research field of using LIFU to stimulate specific targets of the brain associated to emotional processes, with the anticipation of effects on neurocircuits, neurophysiology, depression/anxiety disorders, mood and mood-associated behaviors is concentrated. Inclusion and exclusion criteria are as follows.

Inclusion Criteria:

(1) Research using depressive/anxious-like animal models where LIFU was applied to modulate the phenotypes and behaviors in preclinical studies.
(2) Research recruiting healthy volunteers, where LIFU was used to modulate emotional processes, along with the collection of subjective/objective emotion evaluations or neurophysiology data.
(3) Studies involving patients with depression (comorbid depression) or anxiety disorders, where LIFU was employed to treat these symptoms.

Exclusion Criteria:

(1) Studies utilizing high-intensity focused ultrasound (HIFU) for capsulotomy or thermal ablation surgery.
(2) Research on the effects of exposure to unfocused ultrasound.
(3) Studies using LIFU to facilitate drug delivery via the blood–brain barrier.
(4) Research involving gene engineering, specifically sonogenetics.
(5) Studies combining LIFU or unfocused ultrasound with microbubble injections for neuromodulation.
(6) Research presented only in the form of conference abstracts.
(7) Studies unrelated to the specified themes of this review.

## 3. Results

The search results are indicated in Section 3.1. Studies of preclinical depressive/anxious-like animal models with LIFU treatment are reviewed in Section 3.2. Studies of LIFU’s emotional modulation effect among healthy participants are reviewed in Section 3.3. Studies using LIFU to treat patients with depression are reviewed in Section 3.4. Studies using LIFU to treat patients with anxiety disorder are reviewed in Section 3.5.

### 3.1. Search Results and Categories

**Figure 2:**
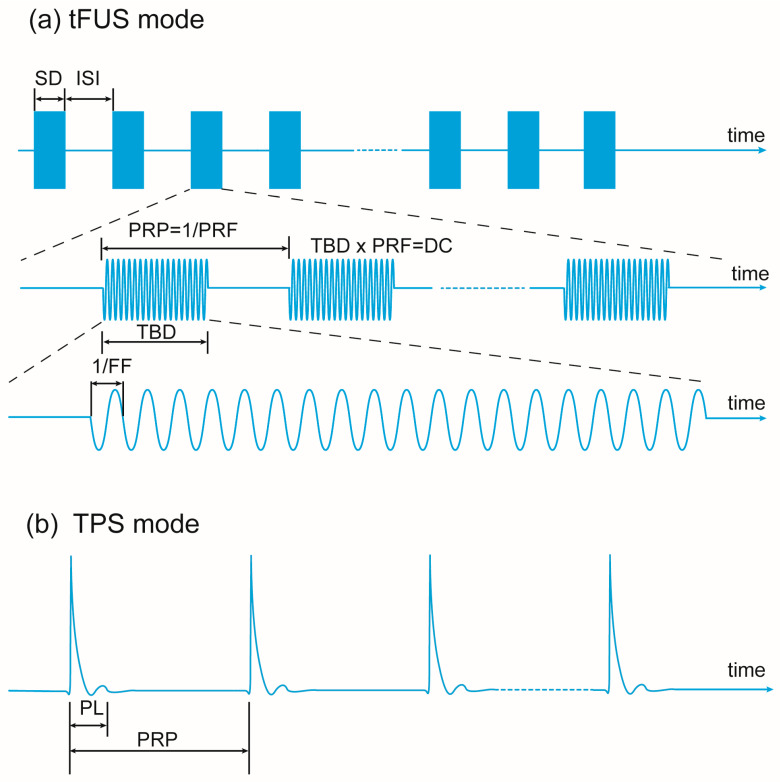
Illustration of parameters for the LIFU technique: (**a**) transcranial focused ultrasound stimulation (tFUS) mode, sonication duration (SD), inter-stimulus interval (ISI), pulse repetition period (PRP), pulse repetition frequency (PRF), tone burst duration (TBD), duty cycle (DC), and fundamental frequency (FF); (**b**) transcranial pulse stimulation (TPS) mode and pulse length (PL).

**Figure 3:**
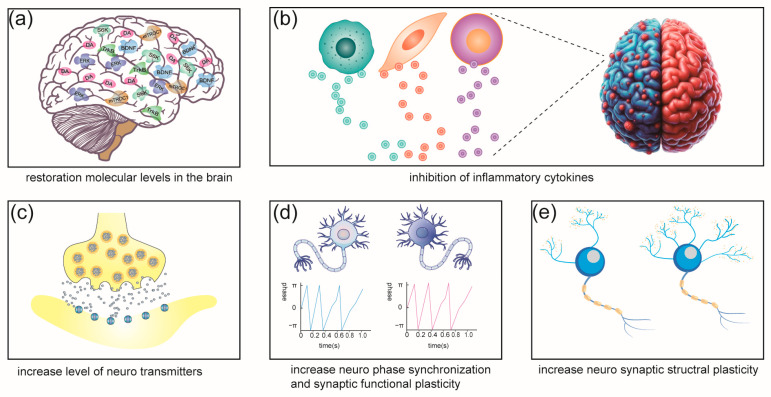
Mechanism found in the current research of LIFU’s mood effects. (**a**) Key molecule restoration after LIFU treatment, including BDNF, DA, ERK, mTROC1, S6K, and TrkB. (**b**) Inflammatory cytokines inhibited after LIFU treatment, including IL-6, IL-1β, and TNF-α. (**c**) Level of neurotransmitters increased after LIFU treatment, including DA, 5-HT, and NE. (**d**) Phase synchronization of neurons increased after LIFU treatment, e.g., the phase locking value (PLV). (**e**) Neurosynaptic structural plasticity increased after LIFU treatment, e.g., number of dendrites increased after LIFU treatment.

**Figure 4:**
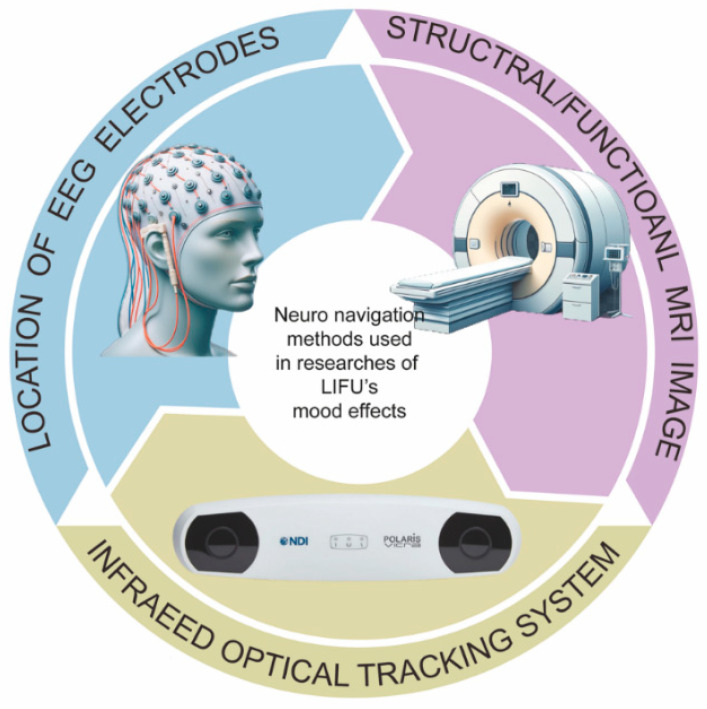
Neuronavigation methods used in research studying the mood modulation effects of LIFU.

Our search yielded 185 papers, of which 28 met the inclusion and exclusion criteria. They were categorized into the flowing four categories: 8 studies on LIFU preclinical experiments in depressive/anxious-like animal models; 6 studies examining the emotional effects of LIFU in healthy volunteers; 12 studies assessing the treatment effects of LIFU in patients with depression or comorbid depression symptoms; 2 studies investigating LIFU treatment effects in patients with anxiety disorders. The papers are reviewed according to their categories and in chronological order. Two different LIFU stimulation modes and the main parameters of the stimulation protocol are demonstrated in Figure 2. The mechanisms found in the current research on LIFU’s mood effects are demonstrated in Figure 3, and the neuronavigation techniques used in LIFU research are shown in Figure 4.

### 3.2. LIFU Preclinical Studies of Depressive/Anxious-like Animal Models

Before clinical application, it is essential to establish animal models to confirm the potential antidepressant effects of LIFU and underlying mechanisms. In 2019, Zhang et al. pioneered to investigate the antidepressant effects of LIFU on a depressive-like rat model with restraint stress method [44]. The left prelimbic cortex (l-PLC), an area homologous to the human prefrontal cortex, was targeted. Behavioral tests, such as the sucrose preference test (SPT), open field test (OFT), and forced swimming test (FST), were conducted, showing improvements in SPT and OFT but not in FST. The study noted a significant increase in BDNF levels in the left hippocampus compared to control groups, leading the authors to hypothesize that LIFU’s antidepressant effects are mediated by stimulating l-PLC to increase hippocampal BDNF levels via PLC–hippocampus excitatory pathways.

In 2020, Zhang et al. developed another depressive animal model using chronic unpredictable stress (CUS), demonstrating that LIFU to the ventromedial prefrontal cortex (vmPFC) could reverse depressive-like behaviors and significantly alter molecular indicators such as BDNF, TrkB, ERK, mTORC1, and S6k [45]. This study highlighted the neuromodulation effects of LIFU on the BDNF/ERK/mTORC1 signaling pathway through vmPFC.

In 2022, Yi et al. developed a depressive mouse model by injecting lipopolysaccharide (LPS), and reported that LIFU to the prefrontal cortex significantly reduced inflammatory cytokine (including IL-6, IL-1β, and TNF-α) expression, improving depression and anxiety behaviors as assessed by behavioral tests such as the OFT, tail suspension test (TST), and elevated plus maze (EPM) [46]. This research indicated that LIFU might ameliorate depressive-like behavior by reducing brain inflammation.

Compared with other non-invasive neuromodulation techniques, like TMS and tDCS, LIFU offers superior penetration depth and spatial resolution, facilitating the direct modulation of subcortical nuclei related to mood processing. Zhu et al. conducted a research utilizing LIFU to target the dorsal raphe nucleus (DRN), a key serotonin source, in a chronic restraint stress (CRS) model [47]. They developed a wearable ultrasound transducer capable of stimulating the DRN in freely moving mice without the need for anesthesia. Their findings indicated that LIFU significantly increased the expression of c-Fos-positive cells and serotonin (5-HT) levels in the DRN area. Behavioral assessments using the SPT and TST revealed improvements in depressive-like behaviors, such as anhedonia and hopelessness. This research supports the potential of LIFU as a novel therapeutic approach for depression through the reversal of monoamine depletion. Recognizing that multiple cerebral areas are implicated in the pathology of depression, the same group reported another study targeting both PLC and DRN to treat depressive-like behaviors in freely moving mice in a CRS model [48]. Compared to the single-target stimulation group, the simultaneous stimulation of PLC and DRN resulted in more pronounced increases in BDNF expression in PLC and serotonin levels in DRN, suggesting that multi-target LIFU treatment may offer enhanced antidepressant effects.

In 2023, Wu et al. established a depressive–anxious comorbidity model of mice through the subcutaneous injection of corticosterone and targeted the DRN with LIFU [49]. Mice in the experimental group received 30 min of LIFU treatment daily for three weeks, while corticosterone injections continued to simulate ongoing stress exposure. Behavioral tests, including SPT, FST, and EPM, demonstrated significant improvements in both depression-like and anxiety-like behaviors compared to control groups. LIFU effectively activated neurons in the DRN, as confirmed by c-Fos staining. On the molecular level, LIFU significantly decreased norepinephrine (NE) levels and slightly increased 5-HT concentrations, suggesting that the antidepressive and anxiolytic effects of LIFU may operate through the restoration of 5-HT and NE concentrations in the brain.

The parameters of LIFU offer a wide range of combinations, and several studies have demonstrated that different LIFU parameters can produce varied neural effects [50,51,52,53,54,55,56,57]. Wang et al. conducted the first study to examine the impact of varying ultrasound intensities on depressive-like behaviors in rats [58]. They targeted l-PFC using two intensity levels of LIFU—I_spta_ = 500 mW/cm^2^ and 230 mW/cm^2^—across different groups. Following the establishment of the CUS depressive model, LIFU treatment was administered for 15 min daily over two weeks. Subsequent assessments using the OFT and EPM indicated that neither intensity level significantly influenced anxiety-like behaviors in the CUS model rats. However, depressive-like behaviors, assessed via the SPT and FST, showed that both intensities had a significant antidepressant effect by reducing anhedonia and despair. No significant differences were found between the two intensity groups. To investigate the underlying mechanisms, changes in synaptic functional plasticity, structural plasticity, and synaptic proteins in the medial prefrontal cortex (mPFC)–vCA1 pathway were examined. Electrophysiological data suggested that the antidepressant effect of LIFU might be associated with increased phase synchronization of the theta band between the vCA1 and mPFC, indicative of enhanced synaptic functional plasticity. Additionally, dendritic spine density significantly increased in the CUS + LIFU group compared to the CUS group alone, suggesting improved synaptic structural plasticity in the mPFC. The expression of synaptic-related proteins, specifically NR2B and PSD95, was significantly elevated in the LIFU-treated group, indicating that LIFU enhanced the expression of postsynaptic proteins in the mPFC of CUS model of rats. In 2024, researchers from the same group conducted another experiment to evaluate LIFU’s mood effect in a CRS-induced depressive model of female mice [59]. The energy of LIFU was targeted at the ventral tegmental area (VTA), and a dopamine (DA) probe was implanted into mPFC to monitor fluctuation of the DA signal in vivo. The c-Fos test confirmed that more neurons in the VTA region were activated in the LIFU group compared to the sham group. Through behavioral assessments, improvements in antidepressive behaviors, such as anhedonia and despair, were observed following LIFU treatment. The DA concentration in mPFC was increased with LIFU, and this increase was positively correlated with the amelioration of depression-like behaviors in the SPT test. Furthermore, histological examinations in this research indicated that LIFU exhibited neuroprotective effects against CRS exposure in both VTA and mPFC regions.

The parameters and main findings of the papers that studied antidepressive/anxious effects of LIFU through animal models are listed in Table 1.

### 3.3. Mood Effects of LIFU Among Healthy Participants

While preliminary results from Hameroff’s pilot research indicated the mood-altering effects of ultrasound [31], several limitations were identified:

(1) The ultrasound energy was delivered by a diagnostic ultrasound system in scan mode and resulted in an unfocused ultrasound beam that lacked precise targeting of brain regions, making it challenging to confirm the affected neural circuits.
(2) Patients participating in the research were enrolled based on symptoms of chronic pain rather than mood disorders, making it difficult to determine whether mood improvements stemmed from direct emotional effects or reductions in pain sensation.
(3) Quantitative data records to evaluate functional changes in the brain resulting from ultrasound stimulation were lacking.

To address these deficiencies, Sanguinetti et al. conducted the first study to investigate the mood effects of LIFU among healthy volunteers in 2019 [60]. A region in the right ventrolateral prefrontal cortex (r-VLPFC), specifically the right inferior frontal gyrus (r-IFG), was targeted for stimulation. The targeting location was determined based on the F8 electrode location of an EEG system. Mood states of participants were assessed using visual analog mood scales (VAMSs). The results revealed that, after 30 s of 500 kHz LIFU treatment to the r-IFG, participants’ mood significantly improved after 20 and 30 min, indicating that positive mood effects persisted for at least 30 min post-LIFU. The researchers utilized fMRI to examine alterations in brain activity associated with the r-IFG post-LIFU treatment. Seed regions of interest included the r-IFG and two major hubs in the Default Mode Network (DMN): mPFC and posterior cingulate cortex (PCC). Functional connectivity (FC) within the r-IFG network decreased significantly after LIFU, particularly between the subgenual cortex, orbitofrontal cortex, inferior prefrontal gyrus, dorsal anterior cingulate cortex, and entorhinal cortex. Conversely, FC between the r-IFG and the right middle frontal gyrus (r-MFG) increased significantly. The DMN network exhibited a decline in FC post-LIFU treatment. These results demonstrated that a 2 min LIFU session on the r-IFG modulated FC within the r-IFG and DMN for at least 20 min, seemingly moving in a direction opposite to mood disorder patterns [61]. The fMRI findings aligned with the VAMS results, illustrating improvements in emotional state.

In 2020, Fini and Tyler reported two consecutive studies on LIFU’s ability to alter conflict resolution, emotion processing, as well as consequential physiology and performance changes [62,63]. In the first study, they observed significant changes in neurophysiological conflict, emotional processing, sustained attention, and cognitive control following stimulation of the dorsal anterior cingulate cortex (dACC) with MR-guided LIFU [62]. Additionally, heart rate variability (HRV), event-related potentials (ERPs), delta, theta, and alpha bands of EEG signals, and DMN activity were significantly altered by LIFU. Results from the Positive and Negative Affect Scale (PANAS) mood questionnaires indicated substantial acute mood changes compared to the sham group, suggesting altered emotional processing and enhanced sustained attention by dACC-targeted LIFU. In the second study, the right anterior insula/frontal operculum (AINS/FO) area was targeted. Significant alterations were observed in fear response, HRV, emotional distraction interference performance, ERPs, and EEG signals across multiple frequency bands (delta, alpha, beta, and theta). These studies demonstrate that MR-guided LIFU can selectively target specific brain regions, modulate larger brain functional networks associated with them, and induce cognitive, attentive, and emotional effects.

Recognizing that learned helplessness tasks can be used as a model for depression-like symptoms, in 2023, Forster et al. designed the first research to study the effects of LIFU on a learned helpless task [64]. In this study, 55 healthy participants were recruited and divided into four groups. The findings revealed that LIFU targeted at the right dorsal lateral prefrontal cortex (r-DLPFC) could significantly suppress midline theta activity and affect cingulate cortex activity. Participants who received LIFU demonstrated superior performance in the learned helplessness task, attributed to enhanced control perception and motivated behavior. Additionally, the LIFU group exhibited lower levels of depressive symptoms compared to the control group. This study suggests that individuals with depression and learned helplessness tendencies may benefit from LIFU treatment, although further research is necessary. Notably, the neuromodulation effects of the offline LIFU lasted for a minimum of 30–90 min, implying that a brief exposure to LIFU can yield enduring positive mood effects.

Following this, Ziebell et al. designed a virtual T-maze task paradigm and enlisted 152 healthy participants to investigate the effects of LIFU on physiology, emotions, behavior, and cortical activity within this context [65]. The stimulation target was the right prefrontal cortex (r-PFC), and subjective mood states were assessed using Self-Assessment Manikin (SAM) and VAMS. The results indicated a significant suppression of midfrontal theta activity (MFT) by LIFU, leading to increased approach behavior and reduced withdrawal behavior in the virtual T-maze task. Consequently, LIFU applied to the r-PFC appeared to demonstrate anti-anxiety effects through MFT inhibition. However, statistical analysis revealed significant mood changes were independent from LIFU, which is possibly due to the infeasibility of recording immediate mood alterations post-treatment within 30 min after LIFU or the strong situational manipulation effects of the T-maze paradigm. It is notable that the offline effects of LIFU in this study persisted for a minimum of 50 to 100 min, underscoring a prolonged offline impact.

In another separate study, Chou et al. investigated the effects of LIFU targeting the left amygdala (l-amygdala) in a fear-inducing task with a cohort of 30 healthy individuals [66]. The simulation model of the sound field indicated the highest average acoustic intensity was distributed in the l-amygdala compared to other subcortical brain regions. The results demonstrated that LIFU application reduced the activation of the l-amygdala, hippocampus, and dACC. Resting-state functional connectivity (rsFC) analysis revealed a decrease in the rsFC of the amygdala–insula, suppression of the rsFC in the amygdala–hippocampus, and a significant increase in the rsFC of the amygdala–vmPFC in the LIFU group compared to the sham group. Correlational analysis indicated a reduction in anxiety ratings among the LIFU group, correlating with diminished activation in the l-amygdala, and also decline in rsFC between the amygdala and insula. This study provides evidence of the anti-anxiety effects achieved by targeting the l-amygdala with LIFU and demonstrates the precise subcortical neuromodulation capacity of LIFU.

The parameters and main findings of the papers that studied the mood modulation effects of LIFU among healthy participants are listed in Table 2.

### 3.4. Studies on Clinical Depression Treatment with LIFU

Although the mechanism behind the antidepressant effect of LIFU is not fully understood, several research groups are committed to integrating this innovative technique into clinical practice.

In 2020, Reznik et al. recruited 24 college students with mild-to-moderate depression symptoms and conducted a double-blind pilot study using LIFU as a five-day intervention method [67]. The LIFU stimulation target was r-IFG. As a pilot study investigating LIFU’s impact on human participants with depression, the researchers selected conservative intensity parameters: I_SPTA_ = 74 mW/cm^2^ and I_SPPA_ = 14 W/cm^2^ with a 30 s exposure duration per session. Findings revealed that individuals who received active LIFU experienced improvements in mood states of worry and increased feelings of happiness compared to the placebo group. This study demonstrated that patients with depressive symptoms might derive emotional benefits from repeated LIFU treatments. However, the severity of depression and anxiety did not diminish after the LIFU intervention. Global mood changes were observed at 10 min post-LIFU treatment, but not at 30 min. The authors attributed the transient mood effects and the lack of efficacy against depression and anxiety in this study to the lower ultrasound intensity utilized.

Li et al. recruited 60 patients with Major Depressive Disorder (MDD) and divided them into experimental and control groups [68]. Patients in the experimental group received treatment combining medication with TMS and MRI-guided LIFU, while those in the control group were treated with medication and TMS alone. The brain stimulation targets in the LIFU procedure were the lateral orbitofrontal cortex, cuneiform lobe, and dorsal prefrontal cortex. Patients’ mental status changes were assessed using the Hamilton Rating Scale for Depression (HRSD), Geriatric Depression Scale (GDS), and the Pittsburgh Sleep Quality Index (PSQI). Both groups showed improvement post-treatment, but the experimental group exhibited significantly better rates of effectiveness, with more substantial improvements in depression and sleep quality. The results indicated that combining TMS and LIFU treatment might bring more benefits to MDD patients.

Beisteiner et al. introduced a novel LIFU modality, termed transcranial pulse stimulation (TPS), and investigated its therapeutic effects on Alzheimer’s Disease (AD) patients [69]. The navigation facilities of the TPS system mainly comprised an infrared camera and an MRI-based neuronavigation subsystem, enabling real-time tracking of each TPS pulse during stimulation. Multiple stimulation targets were selected, including the bilateral frontal cortex, bilateral lateral parietal cortex, and extended precuneus cortex. Significant neuropsychological and memory enhancements were observed among AD patients. Considering depression as a prevalent comorbidity of AD, depression symptoms of the patients were assessed post-TPS treatment using GDS and Beck Depression Inventory (BDI) scales. Notably, scores from these assessments indicated sustained improvement in depression symptoms for up to three months. Additionally, findings from post-TPS treatment patient questionnaires revealed that 26% reported mood improvement, 71% reported stable moods, while only 3% reported worsened mood conditions. In 2022 the same research group conducted a sub-analysis of the previous one, from which the fMRI and neuropsychological data of 18 AD patients were analyzed to further elucidate the antidepressant effect of TPS [70]. According to the statistical analysis, the FC value between the left frontal orbital cortex (l-Forb) and right anterior insula (r-AINS) had a positive correlation with BDI-II depression scores. In 15 out of 18 patients, FC values between l-Forb and r-AINS decreased, coinciding with a significant improvement in depressive symptoms, as indicated by BDI-II scores post-TPS interventions. This connectivity shift between the ventromedial network (VMN) and the salience network (SN) tended towards normalization following TPS treatment. The findings from both the BDI-II results and fMRI data supported the conclusion that TPS treatment exerts an antidepressant effect on AD patients with comorbid depression.

In 2022, Cont et al. reported a retrospective analysis of 11 AD patients with mild to severe symptoms who underwent TPS treatment at a hospital in Kempen, Germany [71]. Assessment of mood states before and after TPS treatment revealed significantly improved depressive symptoms and cognitive functions in the AD patients, as measured by the Alzheimer’s Disease Assessment Scale (ADAS).

In 2024, Riis et al. developed a phased array LIFU system (126-elements phased array positioned on each side of the hemispheres) and named it Diadem [72]. This device delivers focused ultrasound through temporal and parietal skull windows to maximize transmission energy. Through-transmit measurements were utilized to evaluate the attenuation, phase shifts, and focus distortion caused by the skull, which were then compensated for aberrations. A mechanical registration method was proposed to accurately target deep brain areas within or outside the MRI system. The average relative positioning error between fiducial markers on the subjects’ heads and the transducers was 1.64 mm. This method requires only one MRI scan for registering the LIFU device to the brain MRI anatomy image throughout the treatment. Two patients with a history of severe treatment-resistant depression (TRD) were recruited and treated using this device, targeting specific areas in subgenual cingulate cortex (SGC) and right ventral striatum. It is worth noticing that an active sham condition was developed in this study: using the same waveform and emission voltages in an unfocused and planar waveform to cover auditory and sensorial artifacts of the system. Significant improvements in self-reported depression and anxiety were observed after 60 s or longer of LIFU treatment to the SGC compared with the active sham condition. Subsequently, the same research group published another case report to study the durable neuromodulation effects of LIFU on a severe TRD patient [73]. Stimulation targets encompassed three areas associated with the subcallosal cingulate cortex (SCC), including the posterior SCC, anterior SCC, and pregenual cingulate. The BOLD signal of fMRI was acquired simultaneously while LIFU was delivered to the SCC. The collected data revealed rhythmic suppression of the relative BOLD signal by LIFU in the target area, while this phenomenon was absent in the sham condition. The patient experienced remarkable benefits from the treatment, with a significant reduction in the HRSD-6 score from 11 to 0 on the following day and a satisfied self-report. The remission period persisted for at least 44 days, with signs of depression recurrence emerging 5 months post-treatment. Both rapid and sustained antidepressant effects of the LIFU treatment were demonstrated in this case report.

In 2024, Oh et al. recruited 40 MDD patients for a randomized, double-blind, sham-controlled clinical trial to evaluate the safety and efficacy of LIFU in treating depression [74]. The l-DLPFC was selected as the target area. The results demonstrated significant improvement in Montgomery–Asberg Depression Rating Scale (MADRS) scores, and increased FC between the subgenual anterior cingulate cortex (sgACC) and associated brain regions, including the mPFC, cerebellum, middle frontal gyrus, caudate, and orbitofrontal cortex, in the LIFU group compared to the sham group. This clinical trial provided evidence that LIFU treatment can reduce depressive and anxiety symptoms, alleviate suicidal ideation, and modulate FC in targeted brain areas among MDD patients. Fan et al. presented a case report employing dual-phased array crossbeam LIFU to treat a 46-year-old male TRD patient [75]. The stimulated brain areas included the ventral capsule (VC), bed nucleus of the stria terminalis (BNST), and anterior nucleus of the thalamus (ANT). The findings indicated that LIFU stimulation of the ANT significantly reduced depressive symptoms and suppressed DMN connectivity. More recently, Riis et al. conducted another randomized, double-blind, sham-controlled clinical trial involving 22 patients with TRD who were randomly assigned to active and sham groups [76]. The SCC was selected as the stimulation target. The results showed an overall effect of decreased activity in the SCC, increased activity in the left ventrolateral prefrontal cortex (l-vlPFC) and right superior temporal gyrus, and rapid improvement in depressive symptoms following LIFU treatment. No severe adverse effects were observed during or within 24 h after the treatment. However, two participants experienced significant mood swings and developed worsening of suicidal ideation between 24 and 72 h post-treatment. This study suggested the same procedure of LIFU treatment might cause individual differences in psychiatric symptoms, and a careful long-term follow-up visit is required in LIFU clinical practice.

More recently, Schachtner et al. conducted a clinical trial investigating the use of LIFU targeting DMN for the treatment of depression [77]. Specifically, the researchers employed a 128-element matrix array transducer to generate five sub-foci in the anterior medial prefrontal cortex (amPFC) with the electronically steered technique. The LIFU treatment resulted in a significant reduction in depressive symptoms and repetitive negative thinking (RNT). Moreover, LIFU was associated with a faster therapeutic response compared to traditional interventions. To address the aberration and distortion effects induced by the human skull, Attali et al. proposed a personalized acoustic metamaterial approach designed to correct focal deformation and enhance the precision of LIFU energy delivery [78]. This technique, termed “metalens-based transcranial ultrasound stimulation (mTUS),” demonstrated advantages in both increasing acoustic intensity and reducing targeting offsets, as confirmed by numerical simulations and in vivo measurements. In the mTUS trial, the stimulation target was the intersection of three white matter tracts within l-SCC: the forceps minor (FM), the cingulum bundle (CB), and the uncinate fasciculus (UF). The fMRI analysis revealed a significant increase in FC between the l-SCC and l-DLPFC, and a significant decrease in FC between the l-SCC and r-hippocampal/parahippocampal region. Depressive symptoms in five patients with MDD were significantly alleviated after five days of mTUS treatment, as measured by the MADRS. No serious adverse events were observed during the trial or the 4-week follow-up visit.

The parameters and main findings of papers that used LIFU to treat patients with depression disorders are listed in Table 3.

### 3.5. Studies on Clinical Anxiety Treatment with LIFU

In a study on clinical anxiety treatment with LIFU, Kennedy and colleagues enrolled 25 patients with treatment-refractory Generalized Anxiety Disorder (trGAD) who had received pharmacological treatments, TMS, psychotherapy, cognitive behavioral therapy, and other therapies [79]. They explored trGAD treatment with MR-guided LIFU for the first time, targeting the centromedial nucleus of r-amygdala. The Hamilton Anxiety Inventory (HAM-A) and Beck Anxiety Inventory (BAI) were utilized to assess changes in anxiety levels. The Patient Global Impression–Improvement (PGI-I) scale was used to evaluate subjective clinical status changes in patients. Results from HAM-A and BAI scores indicated a significant reduction in anxiety symptoms. Based on PGI-I scores, 16 out of 25 (64%) trGAD patients experienced substantial benefits from LIFU treatment. Furthermore, 8 out of 25 patients (32%) achieved remission of GAD symptoms, as indicated by a completion HAM-A score < 14. Moreover, many patients in the study reported improvements in anxious symptoms, an experience not encountered with previous treatments.

Most recently, Barksdale et al. led a double-blind, sham-controlled, unblinded single-arm clinical trial to study the neuromodulation effects of repetitive LIFU targeting at l-amygdala [80]. The study included participants with mood, anxiety, and trauma-related disorders (MATRDs), as well as a healthy control (HC) group. Structural MRI was used to guide LIFU application, while task-based fMRI assessed neural activity in the amygdala and other brain regions. The results demonstrated a significant average deactivation of the targeted l-amygdala in the LIFU group compared to the sham group and anger-specific emotion task within subject. Scores on the Mood and Anxiety Symptom Questionnaire–General Distress (MASQ-GD) were significantly reduced and were associated with a greater attenuation of activity in r-amygdala. No severe adverse events were reported throughout the study. These findings provide further clinical evidence supporting LIFU as a feasible intervention for mood-related disorders.

The parameters and main findings of papers that used LIFU to treat patients with anxiety disorders are listed in Table 4.

## 4. Discussion

So far, the current studies exploring the emotional effects of LIFU on depressive/anxiety-like animal models, healthy volunteers, and patients with depression and anxiety disorders have been systematically reviewed.

### 4.1. Discussion on Animal Studies with LIFU

Regarding the animal studies, seven out of the eight studies built depressive-like animal models, while one study built a depressive–anxious comorbidity model. These studies demonstrated significant improvements in anhedonia, despair, and anxiety-like behaviors following LIFU treatment. The variety in animal model construction allowed for the investigation of different underlying neurophysiological mechanisms of LIFU on mood modulation.

Among studies utilizing stress-based depression models (RS, CRS, and CUS), four targeted the cerebral cortex (l-PLC, PFC, vmPFC, and mPFC) [44,45,46,58], revealing effects on various molecules, such as BDNF, ERK, and mTORC1. The downregulation of inflammatory cytokines in the brain indicated the ability to suppress inflammation with LIFU. Electrophysiological recordings indicated brain rhythms, neural circuit activities between cortical regions and subcortical nuclei (e.g., mPFC-vCA1), were affected by LIFU [58]. These studies suggest that LIFU has the ability to modulate activity in cerebral cortex, as well as to affect the subcortical nucleus through neurocircuits indirectly and wider neural networks. Three studies targeted subcortical nuclei (DRN, PLC, and VTA) [47,48,59]. The c-Fos experiment showed the direct activation of neurons in DRN and VTA by LIFU, while changing levels of 5-HT in DRN, BDNF in PLC, and DA in mPFC proved the ability of LIFU to precisely affect molecule levels in the targeted subcortical areas and further downstream areas. Additionally, three studies employed a free-moving style of LIFU application with lightweight head-mounted transducers [45,47,49], aiming to eliminate anesthesia-related interferences.

While current studies mainly utilized rodent models (mice and rats), future research could consider building different depressive/anxiety-like models, or expanding to other species such as non-human primates for comprehensive insights. Notably, all animal studies reported no brain tissue damage (hemorrhage, edema, etc.), thereby providing a preliminary indication of the safety of the LIFU procedure.

### 4.2. Discussion on LIFU’s Mood Effects Among Healthy Volunteers

Studies involving healthy volunteers confirmed LIFU’s capacity to induce positive mood effects [60,64,66] or alter human performance in specific tasks [62,63,64,65,66]. LIFU was shown to modulate brain networks (DMN) [60] and FC associated with the target areas (rIFG–rMFG, amygdala–insula, amygdala–hippocampal, and amygdala–vmPFC) [60,66]. Physical responses (EEG, ERPs, and HRV) exhibited alterations under LIFU stimulation [62,63,64,65] and may serve as physiological response indicators. Furthermore, correlations were found between mood effects and the activation of target areas or changes in FC between brain regions [66], suggesting a causal relationship between mood effects and neuromodulation impacts of LIFU. Short LIFU sessions (2 min) among healthy participants were observed to induce long-lasting offline effects ranging between 20 and 100 min [60,64,65], highlighting the need for further investigation into the duration and attenuation trends of these emotional effects. The studies of healthy volunteers also demonstrated LIFU’s effective modulation of brain activity in both cerebral cortex and subcortical areas. However, due to single short-period session set in these studies, further research is needed to explore the emotional effects of periodic LIFU sessions among healthy people.

### 4.3. Discussion on LIFU’s Treatment Study Among Patients with Depression/Anxiety

Significant antidepressant effects were observed in both depression patients [67,68,72,73,74,75,76] and those with Alzheimer’s Disease comorbid with depression [69,70,71], as evaluated using various mood scales and self-reports. Additionally, improvements were noted in anxiety, worry, and sleep quality among the patients. The parameters utilized varied widely across studies; for instance, I_SPTA_ ranged from 71 mW/cm^2^ to 10.6 W/cm^2^, I_SPPA_ ranged from 14 W/cm^2^ to 50.2 W/cm^2^, SD ranged from 30 s to 1 min, and PRF ranged from 4 Hz to 500 Hz. Considering the large parameter space, comprehensive investigation into the effects of different parameter combinations on mood effects remains lacking. There may exist individual response thresholds for ultrasound intensity, where levels below the threshold may fail to induce antidepressant effects or only produce short-term mood effects [67,69,71]. I_SPTA_ and I_SPPA_, representing maximum pulse intensity in spatial or temporal terms, respectively, are common representations of LIFU intensity, potentially linked to various bioeffects. Hence, it is recommended that future research reports both kinds of ultrasound intensities so to enhance the understanding of the fundamental mechanism.

Almost all of the studies adhered to ultrasound parameters within the FDA limits for acoustic diagnostic ultrasound, ensuring I_SPTA_ ≤ 720 mW/cm^2^, I_SPPA_ ≤ 190 W/cm^2^, and Mechanical Index (MI) ≤ 1.9. One study used I_SPTA_ = 10.6 W/cm^2^ but reported no side effects [75]. Meanwhile, most of the reported side effects from studies in this review remained mild [68,69,71], offering preliminary evidence of the safety with the parameters utilized. One study reported mood swing cases from two patients within 24–72 h after LIFU treatment [76], indicating that clinical monitoring is still necessary after the LIFU treatment for several days. Protocol variations were notable; for instance, stimulation periods per session ranged from 30 s to 2 h, and treatment durations varied from 1 day to 2 months. These protocol differences may influence offline effects, ranging from 10 min [67] to more than several months [69,73]. Therefore, optimizing protocols with a comprehensive understanding of the parameters and offline effects about LIFU is essential for future studies. By employing fMRI techniques, direct modulation by LIFU was observed in FC associated with target areas, correlated with improvements in depression. This provides objective evidence of LIFU’s antidepression effects.

While LIFU effectively modulates brain areas in both the cerebral cortex and deep brain regions, the ideal LIFU approach for benefiting patients with mood disorders remains under exploration. Novel LIFU treatment approaches, like LIFU-TMS combined therapy [68], TPS [69,71], phased array LIFU delivery [72,73], and multi-target LIFU stimulation [68,69,70,71,72,73], have demonstrated promise in depression treatment. Future studies should further explore these techniques in larger groups and conduct comparative analyses. Tough two pilot studies [79,80] has showed LIFU might be able to treat anxiety disorders, more research of clinical trial on LIFU’s anti-anxiety effect are still needed in the future.

It is always challenging to describe emotion or depression/anxiety level of individuals quantitatively. General scales such as GDS, HRSD, MADRS, BAI can be used to evaluate the basic emotional level and that after LIFU treatment or longer effects. On the other hand, modern brain monitoring techniques, such as fMRI and EEG, are essential to record the activity of brain areas relating to emotion generation, regulation, and perception before, during, and after the LIFU procedure in order to depict the emotional effects from the brain network level.

### 4.4. Other Considerations

There have been reports that auditory or other sensory effects of the LIFU device may add artifacts to the results [81,82,83]. However, these artifacts are not able to fully account for the FC changes associated with the stimulating targets and long-lasting offline effects. Sham groups should be implemented cautiously to mitigate potential confounding factors and enhance the understanding of LIFU’s mood modulation mechanisms [72,73]. Target selection during the LIFU procedure is crucial for treatment arrangement, as different target selections may lead to varied neuromodulation outcomes [69]. While certain subcortical brain nuclei, such as the nucleus accumbens [84,85,86], ventral tegmental area (VTA) [59,87], hippocampus [27,33,88,89], and striatum [90,91,92], can be directly modulated by LIFU, the emotional impacts of LIFU for humans on these areas remain unexplored. Generally, brain regions related to emotions are anatomically symmetrical but show features of lateralization in mood function [93,94,95]; future studies may optimize target selection considering the lateralization function of the brain with anticipated neuromodulation results in order to maximize the benefits of LIFU treatment.

Neuron navigation techniques are essential for LIFU treatment, particularly when targeting deep brain nuclei. Methods such as EEG electrode localization, anatomical/functional MRI, and optical neuro-guidance systems have been discussed in this review (refer to Figure 4). Concurrently, ultrasound imaging-based brain imaging methods have been rapidly developing in recent years [96,97,98,99,100], presenting a potential alternative for dual-mode LIFU procedures in mood modulation. Mood-related biomarkers, such as MFT, which can be significantly altered by LIFU and that also indicate mood or behavioral changes [64,65], could be further incorporated into the development of a closed-loop LIFU mood modulation system. The closed-loop system shall aim to provide precision and customized LIFU neuromodulation therapy based on individual differences.

Recent research suggests that LIFU may also have therapeutic potential for pain [25,101,102,103] and social dysfunctions [104,105,106]. Given that depression and anxiety frequently co-occur with these conditions, applying LIFU to alleviate pain and enhance social functioning might contribute to symptom improvement in depressive and anxiety disorders. Nevertheless, further studies are required to evaluate the efficacy of LIFU in treating such comorbidities.

## 5. Conclusions

Current studies on the mood effects of LIFU in animal models, healthy volunteers, and patients with depression or anxiety disorders have been systematically reviewed. Although current findings indicate preliminary antidepressant and anxiolytic effects, the underlying molecular, cellular, and neurophysiological mechanisms remain largely unclear. The application of LIFU for treating depression and anxiety is still in its early stages in both research and clinical contexts. Advancing knowledge of LIFU’s fundamental effects and mechanisms on mood may accelerate its clinical translation as a novel neuromodulation technique.

The insights gained from current studies should be cautiously translated into clinical practice and further investigated in larger cohorts. The LIFU technique, characterized by non-invasiveness, reversibility, and high spatial resolution across both the cerebral cortex and deep brain areas, holds promise for patients with depression and anxiety disorders. It represents a significant and unique tool that may enhance the psychiatric treatment armamentarium in the future.

## Figures and Tables

**Table 1:** Characteristics of papers which studied antidepressive/anxious effects of LIFU through animal models.

Study	Animal Species	Animal Model	Target	Sonication Parameters	Protocol	Main Results	Behavior Tests	Damage Report
Zhang et al. (2019) [44]	S-D rats (n = 76)	depressive model: RS	l-PLC	FF = 0.5 MHz, SD = 0.4 s, ISI = 3 s, TBD = 0.4 ms, PRF = 1.5 KHz, DC = 60%, P_max_ = 0.38 MPa, I_SPTA_ = 4.55 W/cm^2^, I_SPPA_ = 7.59 W/cm^2^ (without skull)	15 min per day for 2 weeks	DLB↓, anhedonia↓, exploratory behavior↑; BDNF in left hippocampus↑	SPT, OFT, FST	none
Zhang et al. (2020) [45]	S-D rats (n = 30)	depressive model: CUS	vmPFC	FF = 0.8 MHz, SD = 1 s, ISI = 3 s, TBD = 0.2 ms, PRF = 0.2 KHz, DC = 4%, P_max_ = 0.28 MPa, I_SPTA_ = 248 mW/cm^2^, I_SPPA_ = 6.2 W/cm^2^ (without skull); I_SPTA_ = 154 mW/cm^2^, I_SPPA_ = 3.84 W/cm^2^, MI = 0.28 (with skull)	20 min per day for 4 weeks	DLB↓, anhedonia↓, despair↓; ERK, mTORC1, S6K, BDNF, TrkB↑	SPT, OFT, FST	none
Yi et al. (2022) [46]	C57 mice (n = 47)	depressive model: lipopolysaccharide-induced	PFC	FF = 0.5 MHz, SD = 60 s, ISI = 120 s, TBD = 5 ms, PRF = 0.1 KHz, DC = 50%, P_max_ = 0.62 MPa, I_SPPA_ = 10.09 W/cm^2^ (without skull)	30 min single session	ALB↓, DLB↓, despair↓; IL-6, IL-1β, TNF-α in PFC↓	OFT, FST, TST, EPM	none
Zhu et al. (2023) [47]	C57 mice (n = 50)	depressive model: CRS	DRN	FF = 1.1 MHz, SD = 1 s, ISI = 1 s, TBD = 0.5 ms, PRF = 1 KHz, DC = 50%, P_max_ = 0.44 MPa, I_SPPA_ = 5.68 W/cm^2^ (with skull)	30 min per day for 2 weeks	DLB↓, anhedonia↓, despair↓; 5-HT/c-Fos in DRN↑	SPT, TST	none
Zhu et al. (2022) [48]	C57 mice (n = 10)	depressive model: CRS	PLC and DRN	FF = 1.1 MHz, SD = 1 s, ISI = 1 s, TBD = 0.5 ms, PRF = 1 KHz, DC = 50%	30 min per day for 1 week	BDNF in PLC↑; 5-HT in DRN↑	\	\
Wu et al. (2023) [49]	C57 mice (n = 24)	depressive and anxious model: corticosterone-induced	DRN	FF = 1.1 MHz, SD = 1 s, ISI = 1 s, TBD = 0.5 ms, PRF = 1 KHz, DC = 50%, P_max_ = 283 KPa, I_SPPA_ = 2.42 W/cm^2^ (with skull)	30 min per day for 3 weeks	ALB↓, DLB↓, anhedonia↓, despair↓; 5-HT, NE↑, c-Fos in DRN↑	SPT, TST, EPM	none
Wang et al. (2023) [58]	S-D rats (n = 57)	depressive model: CUS	mPFC	FF = 0.5 MHz, SD = 0.5 s, ISI = 2 s, TBD = 0.3 ms, PRF = 2 KHz, DC = 60%, I_SPTA1_ = 0.5 W/cm^2^, I_SPTA2_ = 0.23 W/cm^2^ (without skull)	15 min per day for 2 weeks	DLB↓; ALB unchanged; anhedonia↓; despair↓ PLV of delta and theta rhythm↑	SPT, FST, EPM	none
Wang et al. (2024) [59]	C57 mice (n = 44)	depressive model: CRS	VTA	FF = 0.5 MHz, SD = 0.2 s, ISI = 1.6 s, TBD = 0.3 ms, PRF = 1.5 KHz, DC = 45%, I_SPTA_ = 150 mW/cm^2^ (without skull); I_SPTA_ = 90 mW/cm^2^ (with skull)	15 min per day for 10 days	DLB↓, anhedonia↓, despair↓; DA level in mPFC↑; increased level of DA in mPFC positively correlated with improved behavior in SPI	SPT, TST, NFT, OFT, EPM, SIT	none

S-D rats: Sprague-Dawley rats; RS: restraint stress; CUS: chronic restraint stress; FF: fundamental frequency; SD: sonication duration; ISI: inter-stimulus interval; TBD: tone burst duration; PRF: pulse repetition frequency; DC: duty cycle; P_max_: maximum pressure; I_SPTA_: spatial peak temporal average intensity; I_SPPA_: spatial peak pulse average intensity; MI: mechanical index; DLB: depressive-like behavior; PLV: phase locking value; ALB: anxious-like behavior; SPI: sucrose preference index; NFT: novelty-suppressed feeding test; SIT: social interaction test; ↑/↓ represents up/down-regulation of the evaluation indicator.

**Table 2:** Characteristics of papers which studied mood modulation effects of LIFU among healthy participants.

Study	Participants	Target	Sonication Parameters	Protocol	Evaluation Methods	Main Results	Navigation Method	Sham or Control Condition Included
Sanguinetti et al. (2020) [60]	experiment 1: healthy volunteers (n = 51) experiment 2: healthy volunteers (n = 9)	r-IFG	FF = 0.5 MHz, PRF = 40 Hz Experiment 1: TBD = 65 us, DC = 0.26%, P_max_ = 1.27 MPa, I_SPTA_ = 130 mW/cm^2^, I_SPPA_ = 54 W/cm^2^, MI = 1.79 (without skull); Experiment 2: TBD = 125 us, DC = 0.5%, P_max_ = 1.26 MPa, I_SPTA_ = 272 mW/cm^2^, I_SPPA_ = 54 W/cm^2^, MI = 1.79 (without skull)	experiment 1: single session 30 s experiment 2: single session 2 min	VAMS, GA, GV, fMRI	experiment 1: 30 s LIFU can induce positive mood effects for at least 30 min; experiment 2: FC in r-IFG↓,FC in DMN↓, FC of r-IFG and r-MFG↑	location of EEG F8 electrode	sham group
Fini et al. (2020) [62]	healthy volunteers (n = 28)	dACC	FF = 0.5 MHz, PRF = 1 KHz, DC = 24%, P_max_ = 1 MPa, I_SPPA_ = 20.4 W/cm^2^ (without skull)	single session 500 ms	PANAS, HRV, EEG	parasympathetic markers of HRV↑, emotional processing altered, sustained attention enhanced, significant effects on ERPs, alteration of alpha/delta/theta band of EEG	MRI + infrared optical tracking system	sham group
Fini et al. (2020) [63]	healthy volunteers (n = 28)	AINS/FO	FF = 1 MHz, SD = 0.5 s, ISI < 2.8 s, TBD = 240 us, PRF = 1 KHz, DC = 24%, P_max_ = 1 MPa, I_SPPA_ = 20.4 W/cm^2^ (without skull)	single session	PANAS, HRV, EEG	fear response/HRV/performance of emotional distraction interference altered; ERP/delta/alpha/beta/theta band of EEG altered	MRI + infrared optical tracking system	sham group
Forster et al. (2023) [64]	healthy volunteers (n = 55)	r-DLPFC	FF = 0.5 MHz, TBD = 125 us, PRF = 40 Hz, DC = 0.5%, P_max_ = 1.09 MPa, I_SPTA_ = 199 mW/cm^2^, I_SPPA_ = 40 W/cm^2^, MI = 1.54 (without skull)	single session 120 s	EEG, ECG, VAMS, BDI-V	offline effects lasted 30–90 min, suppression of midline delta, better than average performance in learned helpless task	location of EEG F8 electrode	sham and control group
Ziebell et al. (2023) [65]	healthy volunteers (n = 152)	r-PFC	FF = 0.5 MHz, TBD = 125 us, PRF = 40 Hz, DC = 0.5%, P_max_ = 1.09 MPa, I_SPTA_ = 199 mW/cm^2^, I_SPPA_ = 40 W/cm^2^, MI = 1.54 (without skull)	single session 120 s	EEG, objective behavioral measurement, SAM	anxiety↓, long-lasting r-PFC TUS effects (50–100 min) on physiology and behavior among large sample	location of EEG F8 electrode	sham stimulation
Chou et al. (2024) [66]	healthy volunteers (n = 30)	l-amygdala	FF = 0.65 MHz, SD = 30 s, ISI = 30 s, TBD = 5 ms, PRF = 10 Hz, DC = 5%, I_SPTA_ = 0.72 W/cm^2^, I_SPPA_ = 14.4 W/cm^2^ (without skull)	single session 20 min	fMRI, subjective anxiety rating scale	anxiety rating↓, amygdala BOLD signal during fear task↓, amygdala activation correlated with decreased subjective anxiety↓	offline MRI with fiducial markers on the transducer	sham group

VAMS: visual analog mood scales; GA: global affect; GV: global vigor; PANAS: Positive and Negative Affect Scale; HRV: heart rate variability; ECG: electrocardiograph; BDI: Beck Depression Inventory; SAM: self-assessment manikin; BOLD: blood oxygen level-dependent; ↑/↓ represents up/down-regulation of the evaluation indicator.

**Table 3:** Characteristics of papers using LIFU to treat patients with depression disorders.

Study	Participants	Target	Sonication Parameters	Protocol	Evaluation Method	Main Results	Side Effects	Navigation Method	Sham or Control Condition Included
Reznik et al. (2020) [67]	college students with mild to moderate depression (n = 24)	r-IFG	FF = 0.5 MHz, P_max_ = 0.65 MPa, I_SPTA_ = 71 mW/cm^2^, I_SPPA_ = 14 W/cm^2^, MI = 0.9 (without skull)	30 s per session, 5 LIFU sessions within 7 days	BDI, OASIS, RRS, and PSWQ	severity of depression and anxiety not reduced, trait worry↓, happiness↑, global affect↑	not reported	EEG F8 location	sham group
LI et al. (2020) [68]	MDD patients (n = 60)	lateral orbitofrontal cortex; cuneiform lobe; dorsal prefrontal cortex	FF = 0.65 MHz, SD = 30 s, ISI = 30 s, TBD = 0.5 ms, PRF = 100 Hz, I_SPTA_ = 720 mW/cm^2^ (without skull)	15 min per session, 5 days in a week for 2 months	HRSD, GDS, PSQI	HRSD↓, GDS↓, PSQI↑	10% adverse reaction (dizziness, vomiting)	fMRI-guided	control group
Beisteiner et al. (2019) [69]	AD patients (n = 35)	center1: dorsolateral prefrontal cortex, bilateral frontal cortex (dorsolateral prefrontal cortex and inferior frontal cortex), bilateral lateral parietal cortex, extended precuneus cortex; center2: global brain stimulation (no specific target)	center1: PL = 3 us, PRF = 4 Hz, EFD = 0.25 mJ/mm^2^, P_max_ = 25 MPa, I_SPTA_ = 0.1 W/cm^2^ (without skull), NOPs = 6000 center2: PL = 3 us, PRF = 5 Hz, EFD = 0.2 mJ/mm^2^, P_max_ = 25 MPa, I_SPTA_ = 0.1 W/cm^2^ (without skull), NOPs = 6000	every ROI stimulated twice per session, 3 sessions per week for 2–4 weeks	CERAD scores, GDS, BDI, fMRI	memory and verbal ability↑; FC for hippocampus, parahippocampal cortex, parietal cortex, and precuneus↑; increased of FC is correlated with improved CERAD scores, LIFU effects lasted for 3 months	4% headache, 3% mood deterioration, no hemorrhages and edema	infrared camera tracking system, MRI-based neuronavigation system	sham stimulation
Matt et al. (2022) [70]	AD patients (n = 18)	bilateral frontal cortex (dorsolateral prefrontal cortex and inferior frontal cortex), bilateral lateral parietal cortex, extended precuneus cortex	PL = 3 us, PRF = 5 Hz, EFD = 0.2 mJ/mm^2^ (without skull), NOPs = 6000	3 sessions per week for 2–4 weeks	BDI-II, fMRI	FC between L-FOrb and R-AIsula decreased, and negative correlation with BDI improvements; FC between VMN and SN trended to be normalized	not reported	infrared camera tracking system, MRI-based neuronavigation system	none
Cont et al. (2022) [71]	AD patients (n = 11)	bilateral frontal cortex, bilateral lateral parietal cortex, extended precuneus cortex, bilateral temporal cortex	PRF = 4 Hz, EFD = 0.20 mJ/mm^2^	6000 pulses per session, six sessions over 2 weeks; or 3000 pulses per session, 12 sessions every day	ADAS, MMSE, MoCA, NRSs	ADAS and ADAS improved, self-reported symptom severity↓, depressive symptoms↓, cognition↑	shown in 4% sessions (pain in jaw, nausea, drowsiness)	MRI-guided system	none
Riis et al. (2024) [72]	MDD patients (n = 2)	SGC, ventral striatum	FF = 0.65 MHz, TBD = 30 ms, PRP = 4.03 s, P_max_ = 1.0 MPa, I_SPTA_ = 0.233 W/cm^2^, I_SPPA_ = 31 W/cm^2^ (without skull), MI = 1.2	single session for 60–180 s	7-point scale, GASE	depression symptom↓, anxiety symptom↓	none	single anatomical MRI scan	sham stimulation
Riis et al. (2023) [73]	severe TRD patient with family history of mood disorders (n = 1)	SCC (posterior SCC, anterior SCC), pregenual cingulate	FF = 0.65 MHz, SD = 1 min, ISI = 1 min, TBD = 30 ms, PRP = 4.03 s, DC = 0.75%, P_max_ = 1.0 MPa	single session treatment: each target was sonicated for 2 min, 10 times in random order	fMRI, HRSD-6, GASE	SCC BOLD signal suppressed; rapid and sustained remission of depressive symptoms	none	single anatomical MRI scan	sham stimulation
Oh et al. (2024) [74]	MDD patients (n = 40)	l-DLPFC	FF = 0.25 MHz, SD = 0.3 s, ISI = 6 s, TBD = 1 ms, PRF = 500 Hz, DC = 50%, P_max_ = 0.3 MPa, I_SPTA_ = 3 W/cm^2^ (without skull)	20 min per session, 6 sessions in 2 weeks (thrice a week)	fMRI, MADRS, QIDS-SR, STAI, SSI, K-POMS	depression↓, anxiety↓, suicidal ideation↓; FC between sgACC and associated brain regions ↑	none	CT/MRI	sham group
Fan et al. (2024) [75]	TRD patient (n = 1)	VC, BNST, ANT	FF = 0.5 MHz, SD = 5.2 ms, ISI = 34.8 ms, PRF = 25 Hz, DC = 13%, P_max_ = 1.169 MPa, I_SPTA_ = 10.6 W/cm^2^, I_SPPA_ = 42.2–50.2 W/cm^2^, MI= 1.654 (without skull)	single session treatment: 300 s per stimulation, 8 stimulations per session with 10 min interval,	VAS, HRSD-6	DMN connectivity ↓; depression↓	none	MRI image	control stimulation (unfocused ultrasound)
Riis et al. (2024) [76]	TRD patients (n = 22)	SCC (anterior, middle, posterior)	FF = 0.65 MHz, SD = 30 ms, ISI = 0.7 s or 1.4 s, TBD = 5 ms, PRF = 100 Hz, DC = 50%, P_max_ = 1 MPa, I_SPPA_ = 31.1 W/cm^2^	2 sessions in a week; one session contained 3 blocks. block A: tolerability test block B: 6 three-minute stimulation block C: 6 three-minute stimulation	PANAS-X, HRSD-6, IDS-SR, GAD-7, fMRI	activity of SCC↓; activity of l-vlPFC, right-superior temporal gyrus↑; depression↓	no severe adverse effects within 24 h; 2 significant mood swings within 24–72 h	MRI	sham group
Schachtner et al. (2025) [77]	MDD patients (n = 20)	amPFC	FF = 400 kHz, TBD = 5 ms, PRF = 10 Hz, P_max_ = 820 kPa, I_SPTA_ = 670 mW/cm^2^ (without skull)	11 sessions in up to 3 weeks	BDI-II, HRSD, PTQ	depression↑; RNT↓; rapid antidepressant effects	no serious adverse events	MRI	none
Attali et al. (2025) [78]	MDD (n = 5)	intersection of 3 white matter tracts in l-SCC: FM, UF, CB	FF = 500 kHz, SD = 5 s, ISI = 10 s, TBD = 4.5 ms, PRF = 14 Hz, DC = 6%, Pmax = MPa, I_SPTA_ = 184.4 ± 74.0 mW/cm^2^ (with metalens), MI =1.61	5 min per session, 25 sessions in 5 days	MADRS, HRSD, QIDS-SR	depression↓; FC between L-SCC and l-DLPFC↑; FC between SCC and right hippocampal, r-parahippocampal region↓	no serious adverse events	MRI, CT, optical neuronavigator system	none

OASIS: Overall Anxiety Severity and Impairment Scale; RRS: Ruminative Responses Scale; PSWQ: Penn State Worry Questionnaire; EFD: energy flux density; NOPs: number of pulses; CERAD: The Consortium to Establish a Registry for Alzheimer’s Disease; MMSE: Mini-Mental Status Examination; MoCA: Montreal Cognitive Assessment; NRSs: Numeric Rating Scales; GASE: The General Assessment of Side Effects; QIDS-SR: Quick Inventory of Depressive Symptomatology-Self Report; STAI: State-Trait Anxiety Inventory; SSI: Scale for Suicide Ideation; K-POMS: Korean edition of the Profile of Mood States; VAS: visual analog scales; PANAS-X: The Sadness sub-scale of the expanded Positive and Negative Affect Schedule; IDS-SR: Inventory of Depressive Symptomatology–Self Report; GAD-7: 7-item Generalized Anxiety Disorder scale; PTQ: Perseverative Thinking Questionnaire; RNT: repetitive negative thinking;↑/↓ represents up/down-regulation of the evaluation indicator.

**Table 4:** Characteristics of papers using LIFU to treat patients with anxiety disorders.

Study	Participants	Target	Sonication Parameters	Protocol	Evaluation Method	Main Results	Side Effects	Navigation Method	Sham or Control Condition Included
Mahdavi et al. (2023) [79]	severe trGAD patients (n = 25)	centromedial nucleus of r-amygdala	FF = 0.65 MHz, SD = 30 s, ISI = 30 s, TBD = 5 ms, PRF = 10 Hz, DC = 5%, P_max_ = 0.61 MPa, I_SPTA_ = 719.73 mW/cm^2^, I_SPPA_ = 14.39 W/cm^2^, MI = 0.75 (without skull)	10 min per session, weekly for 8 weeks	HAM-A, BAI, PGI-I	anxiety symptoms↓, 64% gained significant benefit, 32% achieved remission of GAD	none	functional and structural MRI, optical neuronavigation system	none
Barksdale et al. (2025) [80]	MATRDs patients (n = 29); HC (n = 23)	l-amygdala	SD = 30 s, ISI = 30 s, TBD = 5 ms, PRF = 10 Hz, DC = 5%, P_max_ = 0.64 MPa, I_SPTA_ = 719.91 mW/cm^2^, I_SPPA_ = 14.4 W/cm^2^	10 min per session, 15 sessions daily per week for 3 weeks	MASQ-GD	activity in l-amygdala and r-amygdala↓; MASQ-GD↓	non-severe side effects	MRI, optical neuronavigation system	sham stimulation

trGAD: treatment-refractory Generalized Anxiety Disorder; HAM-A: Hamilton Anxiety Inventory; BAI: Beck Anxiety Inventory; PGI-I: Patient Global Impression–Improvement; MATRDs: mood, anxiety, and trauma-related disorders; HC: healthy control; MASQ-GD: Mood and Anxiety Symptom Questionnaire–General Distress; ↓ represents down-regulation of the evaluation indicator.

## Data Availability

No new data were created or analyzed in this study. Data sharing is not applicable to this article.

## Abbreviations

5-HT	serotonin
ADAS	Alzheimer’s Disease Assessment Scale
ALB	anxious-like behavior
amPFC	anterior medial prefrontal cortex
ANT	anterior nucleus of the thalamus
BAI	Beck Anxiety Inventory
BDI	Beck Depression Inventory
BDNF	brain-derived neurotrophic factor
BNST	bed nucleus of the stria terminalis
CB	cingulum bundle
CERAD	Consortium to Establish a Registry for Alzheimer’s Disease
CRS	chronic restraint stress
CUS	chronic unpredictable stress
DA	dopamine
DLB	depressive-like behavior
dACC	dorsal anterior cingulate cortex
DBS	deep brain stimulation
DC	duty cycle
DMN	Default Mode Network
DRN	dorsal raphe nucleus
EFD	energy flux density
EPM	elevated plus maze
ERPs	event-related potentials
FC	functional connectivity
FF	foundation frequency
FM	forceps minor
FST	forced swimming test
GASE	General Assessment of Side Effects
GDS	Geriatric Depression Scale
HC	healthy control
HIFU	high-intensity focused ultrasound
HRSD/HRSD-6	Hamilton Rating Scale for Depression/6-item short version
HRV	heart rate variability
ISI	inter-stimulus interval
I_SPTA_	spatial peak temporal average intensity
I_SPPA_	spatial peak pulse average intensity
K-POMS	Korean edition of the Profile of Mood States
l-amygdala	left amygdala
l-Forb	left frontal orbital cortex
l-PLC	left prelimbic cortex
l-vlPFC	left ventrolateral prefrontal cortex
LIFU	low-intensity focused ultrasound
LPS	lipopolysaccharide
MADRS	Montgomery–Åsberg Depression Rating Scale
MASQ-GD	Mood and Anxiety Symptom Questionnaire–General Distress
MATRDs	mood, anxiety, and trauma-related disorders
MFT	midfrontal theta activity
MMSE	Mini-Mental Status Examination
MI	mechanical index
MoCA	Montreal Cognitive Assessment
mPFC	medial prefrontal cortex
mTUS	metalens-based transcranial ultrasound stimulation
NE	norepinephrine
NFT	novelty-suppressed feeding test
NOPs	number of pulses
NRSs	Numeric Rating Scales
OASIS	Overall Anxiety Severity and Impairment Scale
OFT	open field test
PANAS/PANAS-X	Positive and Negative Affect Scale/Expanded version
PCC	posterior cingulate cortex
PGI-I	Patient Global Impression–Improvement
PL	pulse length
PLV	phase locking value
PRF	pulse repetition frequency
PRP	pulse repetition period
PSQI	Pittsburgh Sleep Quality Index
PSWQ	Penn State Worry Questionnaire
PTQ	Perseverative Thinking Questionnaire
QIDS-SR/IDS-SR	Quick/Inventory of Depressive Symptomatology, Self Report
r-AINS	right anterior insula
r-DLPFC	right dorsolateral prefrontal cortex
r-IFG	right inferior frontal gyrus
r-MFG	right middle frontal gyrus
r-PFC	right prefrontal cortex
RS	restraint stress
r-VLPFC	right ventrolateral prefrontal cortex
RRS	Ruminative Responses Scale
rsFC	resting-state functional connectivity
SAM	Self-Assessment Manikin
SCC	subcallosal cingulate cortex
SD	sonication duration
sgACC	subgenual anterior cingulate cortex
SGC	subgenual cingulate cortex
SIT	social interaction test
SN	salience network
SPI	sucrose preference index
SPT	sucrose preference test
SSI	Scale for Suicide Ideation
STAI	State-Trait Anxiety Inventory
TBD	tone burst duration
tDCS	direct current stimulation
tFUS	transcranial focused ultrasound stimulation
TMS	transcranial magnetic stimulation
TPS	transcranial pulse stimulation
TRD	treatment-resistant depression
trGAD	treatment-refractory Generalized Anxiety Disorder
TST	tail suspension test
TUS	transcranial ultrasound stimulation
UF	uncinate fasciculus
VAS/VAMS	visual analog scales/visual analog mood scales
VC	ventral capsule
VMN	ventromedial network
vmPFC	ventromedial prefrontal cortex
VTA	ventral tegmental area
